# Circ_0061140 knockdown inhibits tumorigenesis and improves PTX sensitivity by regulating miR-136/CBX2 axis in ovarian cancer

**DOI:** 10.1186/s13048-021-00888-9

**Published:** 2021-10-14

**Authors:** Jun Zhu, Jun-e Luo, Yurong Chen, Qiong Wu

**Affiliations:** 1 Department of Obstetrics and Gynecology, Suizhou Hospital, Hubei University of Medicine, Suizhou Central Hospital, No. 60 Longmen Street, Dongcheng District, Suizhou, 441300 China; 2 Department of Gynecology, Suizhou Hospital, Hubei University of Medicine, Suizhou Central Hospital, Suizhou, 441300 China; 3 Department of Obstetrics and Gynecology, Suizhou Maternal and Child Health Hospital, Suizhou, 441300 China

**Keywords:** CBX2, circ_0061140, miR-136, Ovarian cancer, PTX

## Abstract

**Background:**

Ovarian cancer is an aggressive tumor in women with high mortality. Paclitaxel (PTX) can be used for the chemotherapy of ovarian cancer. Here, the roles of circular_0061140 (circ_0061140) in PTX sensitivity and malignant progression of ovarian cancer are unveiled.

**Methods:**

The expressions of circ_0061140, microRNA-136 (miR-136) and chromobox 2 (CBX2) mRNA were detected by quantitative real-time polymerase chain reaction (qRT-PCR). Protein expression was determined by western blot. The half maximal inhibitory concentration (IC_50_) of PTX was determined by 3-(4,5-Dimethylthazol-2-yl)-2,5-diphenyltetrazolium bromide (MTT) assay. Cell proliferation was investigated by cell counting kit-8 (CCK-8) and colony formation assays. Cell apoptosis was demonstrated by flow cytometry analysis. Cell migration and invasion were evaluated by transwell assay. The binding relationship between miR-136 and circ_0061140 or CBX2 was predicted by interactome or starbase online database, and identified by dual-luciferase reporter assay. The effects of circ_0061140 on tumor formation and PTX sensitivity in vivo were disclosed by tumor formation assay.

**Results:**

Circ_0061140 and CBX2 expressions were upregulated, while miR-136 expression was downregulated in PTX-resistant tissues and cells compared with control groups. Circ_0061140 knockdown repressed cell proliferation, migration and invasion, and promoted cell apoptosis and PTX sensitivity; however, these effects were restrained by miR-136 RNAi. Additionally, circ_0061140 was a sponge of miR-136, and miR-136 bound to CBX2. Furthermore, circ_0061140 knockdown inhibited tumor formation and improved PTX sensitivity in vivo.

**Conclusions:**

Circ_0061140 silencing repressed the progression and PTX resistance of ovarian cancer by downregulating CBX2 expression via sponging miR-136, which provided novel insight into studying the therapy of ovarian cancer with PTX.

**Supplementary Information:**

The online version contains supplementary material available at 10.1186/s13048-021-00888-9.

## Background

Ovarian cancer is a malignant gynecological tumor with high mortality in women worldwide [1, 2]. More than 65% patients are diagnosed at an advanced stage of the disease [3]. Although many processes have been achieved in therapy, the prognosis of ovarian cancer patients is still poor. Paclitaxel (PTX) is a commonly used chemotherapy drug of ovarian cancer; however, its resistance gradually develops during cancer therapy, ultimately leading to the therapeutic failure [4]. Therefore, deeply understanding the mechanism related to PTX resistance is necessary for improving the unfavorable prognosis of ovarian cancer patients.

Circular RNA (circRNA) is a noncoding RNA with closed loop structure and modulates gene expression via sequestering microRNA (miRNA) [5, 6]. Multiple research studies have indicated that circRNA is involved in the pathogenesis of various cancers, including ovarian cancer [7–9]. For example, circ_0051240 facilitated cell proliferation and tumor metastasis via sponging miR-637 in ovarian cancer [10]. Circ_0078607 restrained cell proliferation and facilitated cell apoptosis in ovarian cancer [9]. Besides, considerable evidence has suggested that circ_0063809 silencing and enforced expression of circEXOC6B sensitize ovarian cancer cells to PTX [11, 12]. Previous study has disclosed that circ_0061140 expression is increased in ovarian cancer cells, and its silencing hinders cell proliferation and migration [13]. However, the effects and regulatory mechanism of circ_0061140 in PTX sensitivity of ovarian cancer remain still unknown.

MiRNAs are a category of small noncoding RNAs with about 22 nucleotides (nts) in size; and they regulate gene expression via associating with their 3′-untranslated region (3’UTR) [14]. MiRNAs were involved in many biological processes, such as cell proliferation, metastasis, apoptosis and so on [15, 16]. MiR-136, a miRNA, has been reported to suppress the progression of renal cell carcinoma [17], colorectal cancer [18] and osteosarcoma [19]. Additionally, Zhao et al. suggested that miR-136 repressed cell survival in ovarian cancer [20]. Chromobox (CBX) family plays vital roles in gene expression and developmental programming [21, 22]. Wheeler et al. explained that the member of CBX, CBX2, participated in anoikis escape in high grade serous ovarian carcinoma [23]. The above data demonstrated that both miR-136 and CBX2 might be related to ovarian cancer development.

As predicted by online databases, miR-136 contained the putative binding sites of circ_0061140 and CBX2. Thus, we hypothesized that circ_0061140 regulated PTX sensitivity in ovarian cancer by miR-136/CBX2 pathway. In the present study, we analyzed the effects of circ_0061140 in the progression and PTX sensitivity of ovarian cancer in vitro and in vivo, and demonstrated whether the mechanism underlying the sensitivity of ovarian cancer involved circ_0061140/miR-136/CBX3 pathway.

## Materials and methods

### Tissue collection and storage

PTX-resistant human ovarian cancer tissues (*N* = 20) and PTX-sensitive human ovarian cancer tissues (*N* = 19) were separated from ovarian cancer patients from Suizhou Hospital, Hubei University of Medicine. These tissues were stored at − 80 °C. All study subjects signed the written informed consents. The study was approved by the Ethics Committee of Suizhou Hospital, Hubei University of Medicine and carried out according to the guidelines of Declaration of Helsinki. PTX-resistant ovarian cancer tissues were classified as those with recurrent disease within 6 months after PTX-based chemotherapy. PTX-sensitive tumors were regarded as those with a response to PTX-based chemotherapy and a platinum-free interval of 6 months. The clinical features of study subjects were displayed in Table S2.

### Cell acquisition and the establishment of PTX-resistant cells

Human ovarian cancer cell lines (SKOV3 and HeyA8) and human normal ovarian epithelial cell-line IOSE-80 were purchased from Otwo Biotech (Shenzhen, China). All cells were cultivated in Roswell Park Memorial Institute-1640 (RPMI-1640; HyClone, Logan, UT, USA) supplemented with 10% fetal bovine serum (FBS; HyClone) and 1% streptomycin/penicillin (Millipore, Bradford, MA, USA) at 37 °C in an incubator with 5% CO_2_.

In order to establish PTX-resistant ovarian cancer cells, SKOV3 and HeyA8 cells were treated with the 1/50 half maximal inhibitory concentration (IC50) of PTX (Millipore) and cultured until cells stably grew. Then, cells were treated with an increasing dose of PTX in multiples. Cells were cultivated for 15 days after treatment of each dose of PTX, which lasted more than 6 months. The induced PTX-resistant ovarian cancer cells were named as SKOV3/PTX and HeyA8/PTX.

### Plasmids construction and cell transfection

Small interfering RNAs targeting circ_0061140 (si-circ_0061140#1 and si-circ_0061140#2), miR-136 mimic (miR-136), the RNA interference (RNAi) of miR-136 (anti-miR-136), the overexpression plasmid of CBX2 (pcDNA-CBX2), small hairpin RNA against circ_0061140 (sh-circ_0061140) and controls (si-NC, miR-NC, anti-miR-NC, pcDNA-NC and sh-NC) were synthesized by GENEWIZ (Suzhou, China). Plasmids were transfected into cells using Lipofectamine 3000 (Invitrogen, Carlsbad, CA, USA). The sequences in this part were si-circ_0061140#1 5′-TTCTCAGAAGTGTGGAGTGAA-3′, si-circ_0061140#2 5′-CAGAAGTGTGGAGTGAAGTTA-3′, miR-136 mimic 5′-ACUCCAUUUGUUUUGAUGAUGGA-3′, miR-136 RNAi 5′-UCCAUCAUCAAAACAAAUGGAGU-3′, si-NC 5′-GGAGATTCTAGTAGGAGAA-3′, miR-NC 5′-UUUGUACUACACAAAAGUACUG-3′ and anti-miR-NC 5′-CAGUACUUUUGUGUAGUACAAA-3′.

### 3-(4,5)-dimethylthiahiazo (−z-y1)-3,5-di-phenytetrazoliumromide (MTT) assay

The viability of PTX-resistant SKOV3 and HeyA8 was analyzed using MTT detection kit (Beyotime, Jiangsu, China). In brief, cells were seeded in 96-well plates (5 × 10^3^ per well) and cultured for 16 h. The cells were treated with test compounds for the defined time, and MTT solution (Beyotime) was added into each culture well. Four hours later, dimethyl sulfoxide (Millipore) was adopted to dissolve formazan. Finally, cell viability was analyzed using microplate reader (Thermo Labsystems, Waltham, MA, USA) with absorbance at 570 nm.

### Quantitative real-time polymerase chain reaction (qRT-PCR)

Ovarian cancer tissues and cells were lysed using TransZol (TransGen Biotech, Beijing, China) and RNA was extracted with an RNAsimple kit (Tiangen, Beijing, China). cDNA was synthesized using a primeScript™ kit (TaKaRa, Shiga, Japan) or Mir-x™ miRNA Kit (TaKaRa). After that, SuperReal PreMix Color (Tiangen) was conducted to detect the expression of circ_0061140, miR-136 and CBX2. Data were analyzed by the 2^-∆∆Ct^ method with U6 and glyceraldehyde 3-phosphate dehydrogenase (GAPDH) as references. The primer sequences were listed in Table S1.

### Cell counting kit-8 (CCK-8) assay

The proliferation of PTX-resistant SKOV3 and HeyA8 cells was detected by CCK-8 kit (Beyotime). Briefly, the cells were grown in 96-well plates for 16 h. After various treatments, the cells were cultured for another 24, 48 and 72 h, respectively. CCK-8 solution (Beyotime) was used to incubate the cells for another 4 h. At last, samples were analyzed using with microplate reader (Thermo Labsystems) with absorbance at 450 nm.

### Colony formation assay

PTX-resistant SKOV3 and HeyA8 cells were seeded in 6-well plates. After 16 h, cells were transfected with plasmids and cultured for 2 weeks. RPMI-1640 medium (HyClone) was replaced every 3 days. Supernatant was discarded and cells were washed using phosphate buffer solution (PBS; Solarbio, Beijing, China). Following that, positive colonies were fixed using paraformaldehyde (Beyotime), and dyed with crystal violet (Millipore). Cell colony-forming ability was assessed via counting colony numbers. A colony was defined when its cell numbers more than 50.

### Flow cytometry analysis

The apoptosis of PTX-resistant SKOV3 and HeyA8 cells was determined using Annexin V-fluorescein isothiocyanate (Annexin V-FITC)/propidium iodide (PI) detection kit (Yeasen Biotech, Shanghai, China). In short, at 48 h after transfection, cells were collected via centrifuging. Cells were mixed with 100 μL binding buffer (Yeasen Biotech). After that, Annexin V-FITC (Yeasen Biotech) and PI (Yeasen Biotech) were employed to dye the cells. Finally, these samples were assessed using flow cytometry (BD Biosciences, San Diego, CA, USA).

### Transwell assay

Transwell chamber without or with Matrigel (Corning, New York, Madison, USA) was employed to detect cell migration and invasion, respectively. Shortly, cells were mixed with serum-free RPMI-1640 medium (HyClone) and then placed into the upper chamber. RPMI-1640 medium containing 15% FBS (HyClone) was added into the lower chamber. After 24 h, medium was discarded, and methanol (Millipore) and crystal violet (Millipore) were used to fix and stain the migrated and invading cells, respectively. Results were analyzed under microscope (Olympus, Tokyo, Japan).

### Dual-luciferase reporter assay

Interactome or starbase online database was performed to predict the binding sites between miR-136 and circ_0061140 or CBX2. Subsequently, the wild-type (WT) plasmids (circ_0061140-WT and CBX2 3’UTR-WT) were built by inserting the sequences of circ_0061140 and the 3′-untranslated region (3’UTR) of CBX2 into psiCHECK2 vector (Hanbio Biotechnology, Shanghai, China). In the same manner, the mutant (MUT) plasmids (circ_0061140-MUT and CBX2 3’UTR-MUT) were constructed. Constructed plasmids were transfected into cells with miR-136 mimic or miR-NC using Lipofectamine 3000 (Invitrogen). Luciferase activities were detected by luciferase reporter assay kit (Promega) with *Renilla* Luciferase activity as a control.

### Western blot

Ovarian cancer tissues and cells were lysed with lysis buffer (Beyotime). The lysates were boiled and mixed with loading buffer (Thermo Fisher). Lysates were loaded onto 12% bis-tris-acrylamide gels (Thermo Fisher), and proteins were transferred onto polyvinylidene fluoride (Millipore). Membranes were immersed in 5% nonfat milk (Solarbio) at 4 °C for 4 h. Following that, these membranes were incubated with the antibodies against GAPDH (ab128915; 1:20000; Abcam) or the long isoform of CBX2 (ab182620; 1:500; Abcam) overnight at 4 °C, respectively. Secondary antibody labeled with horseradish peroxidase (ab205718; 1:5000; Abcam) was used to incubate membranes at 37 °C for 2 h. Protein bands were visualized using enhanced chemiluminescence system (Pierce, Rockford, IL, USA). GAPDH was employed as a reference.

### In vivo tumor formation assay

Five-week-old female BALB/c nude mice were purchased from Charles River (Beijing, China). Nude mice were fed in pathogen-free environment, and randomly divided into 4 groups: sh-NC group, sh-NC + PTX group, sh-circ_0061140 group and sh-circ_0061140 + PTX group (*N* = 5, respectively). SKOV3/PTX cells (1 × 10^6^) transfected with sh-circ_0061140 or sh-NC were subcutaneously injected into the back of mice. After 7 days, mice were intraperitoneally treated with PTX (3 mg/kg) diluted with PBS (Solarbio) every 3 days for 5 times. Tumor volume was measured every 7 days since PTX treatment according to the formula: Volume (mm^3^) = width^2^ × length/2. Twenty-eight days later, mice were killed and tumors were excised for further study of tumor weight and the expression of circ_0061140, miR-136 and CBX2. The study was permitted by the Animal Care and Use Committee of Suizhou Hospital, Hubei University of Medicine and performed in accordance with the guidelines of the National Animal Care and Ethics Institution.

### Statistical analysis

Data from 3 replicates were analyzed using SPSS 21.0 software (IBM, Somers, NY, USA) and expressed as means ± standard deviations (SD). The linear relationship between miR-136 and circ_0061140 or CBX2 was analyzed by Spearman correlation analysis. Significant differences were compared with two-tailed Student’s *t*-tests, Wilcoxon rank-sum test or one-way analysis of variance. *P* value < 0.05 was considered statistically significant.

## Results

### Circ_0061140 expression was dramatically upregulated in PTX-resistant ovarian cancer tissues and cells

**Fig. 1:**
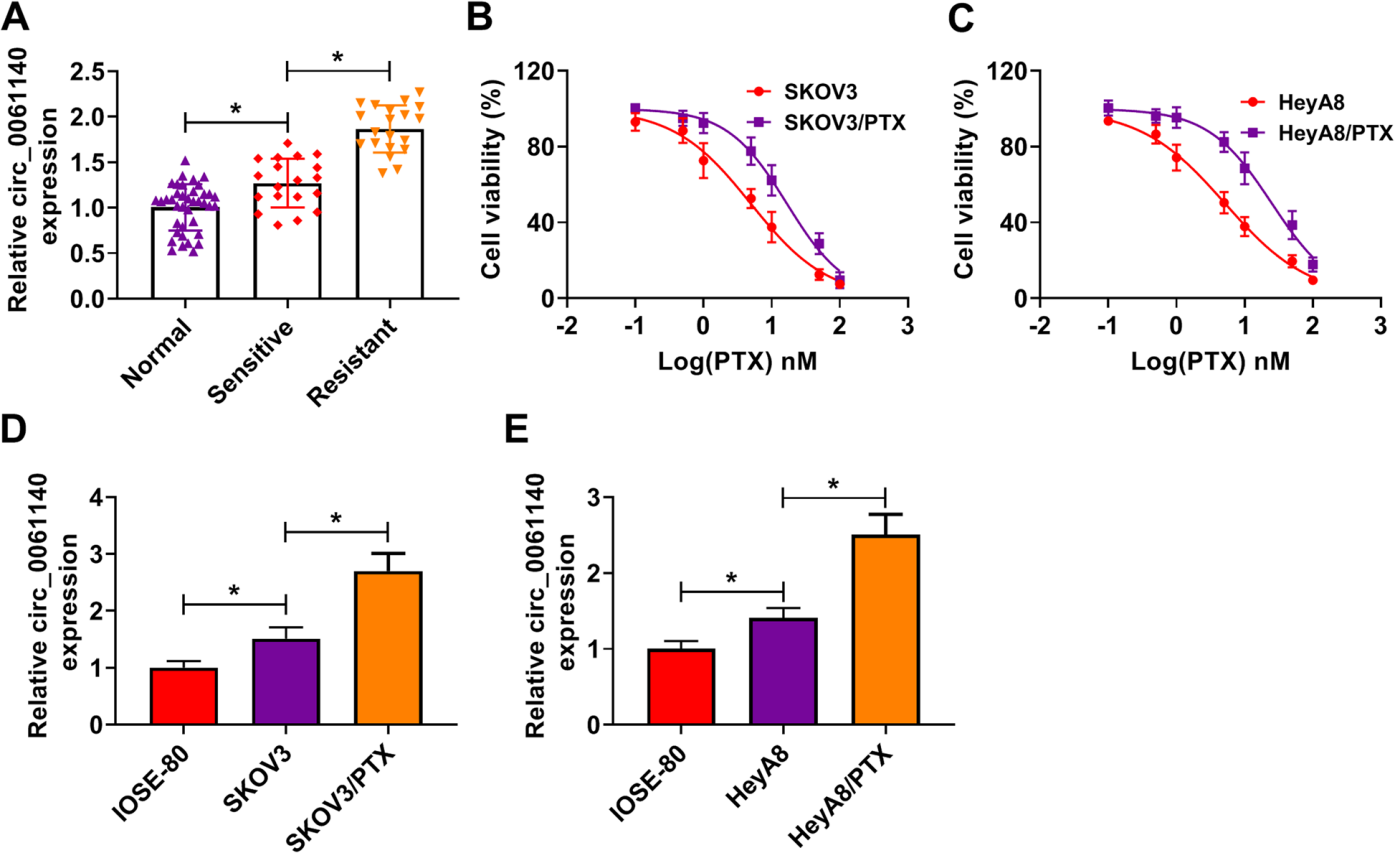
Circ_0061140 was highly expressed in PTX-resistant ovarian cancer cells. (**A**) Circ_0061140 expression was checked by qRT-PCR in normal ovarian tissues (*N* = 39), PTX-sensitive ovarian cancer tissues (*N* = 19) and PTX-resistant ovarian cancer tissues (*N* = 20). (**B** and **C**) MTT assay was employed to detect cell viability after PTX treatment (0.1, 0.5, 1, 5, 10, 50 and 100 nM) in SKOV3 and HeyA8 cells and PTX-resistant SKOV3 and HeyA8 cells. (**D** and **E**) QRT-PCR was performed to determine circ_0061140 expression in IOSE-80 cells, SKOV3 and HeyA8 cells as well as PTX-resistant SKOV3 and HeyA8 cells. *P < 0.05

Circ_0061140 was located in chromosome 20:61576090–61,579,827 and generated from C20orf11 gene. In order to demonstrate whether circ_0061140 participated in regulating the sensitivity of ovarian cancer to PTX, we detected its expression in PTX-sensitive and PTC-resistant ovarian cancer tissues. Results firstly showed that circ_0061140 level was higher in PTC-resistant tissues than in PTX-sensitive tissues (Fig. 1A). To determine the expression of circ_0061140 in PTX-resistant SKOV3 and HeyA8 cells, the IC_50_ of PTX was measured by MTT assay. As shown in Fig. 1B and C, the IC_50_ of PTX was significantly increased in PTX-resistant SKOV3 and HeyA8 cells (Fig. 1B and C), which suggested that PTX-resistant ovarian cancer cells were successfully established. Subsequently, circ_0061140 expression was significantly upregulated in SKOV3/PTX and HeyA8/PTX cells compared with SKOV3 and HeyA8 cells (Fig. 1D and E). These data indicated that circ_0061140 might be related to the sensitivity of ovarian cancer to PTX.

### Circ_0061140 knockdown improved PTX sensitivity and repressed ovarian cancer cell malignancy

**Fig. 2:**
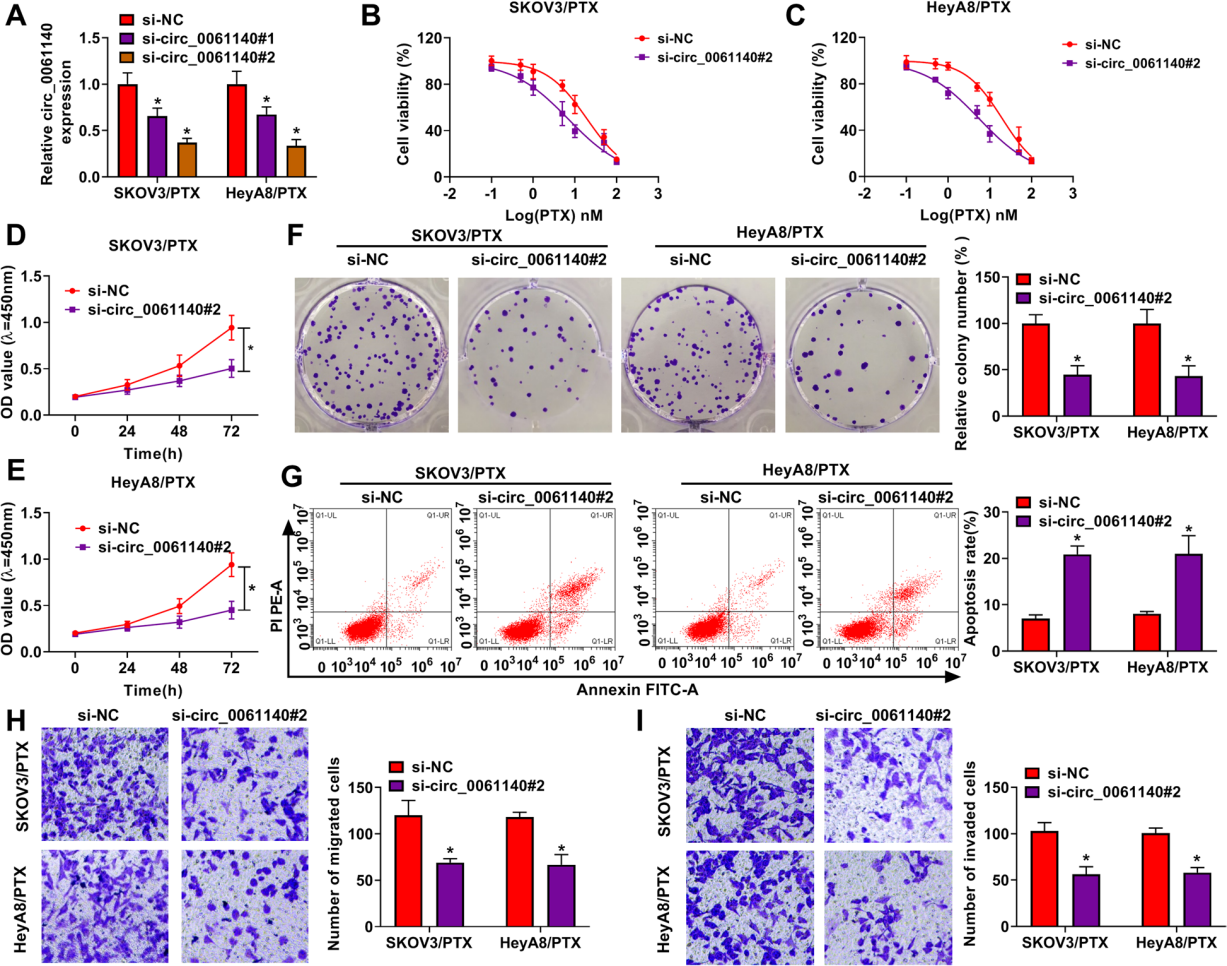
Circ_0061140 silencing repressed the PTX resistance and development of ovarian cancer. (**A**) The knockdown efficiency of circ_0061140 and circ_0061140 was detected by qRT-PCR in PTX-resistant SKOV3 and HeyA8 cells. (**B** and **C**) The effects of circ_0061140 silencing on cell viability after PTX exposure (0.1, 0.5, 1, 5, 10, 50 and 100 nM) were determined by MTT assay in PTX-resistant SKOV3 and HeyA8 cells. (**D**-**F**) The impact of circ_0061140 knockdown on cell proliferation was revealed by CCK-8 and colony formation assays in PTX-resistant SKOV3 and HeyA8 cells. (**G**) Flow cytometry analysis was performed to unveil the impact of circ_0061140 silencing on the apoptosis of PTX-resistant SKOV3 and HeyA8 cells. (**H** and **I**) The influences of circ_0061140 absence on the migration and invasion of PTX-resistant SKOV3 and HeyA8 cells were investigated by transwell assay. *P < 0.05

The study continued to analyze whether circ_0061140 regulated PTX sensitivity in ovarian cancer. We transfected the siRNAs of circ_0061140 into PTX-resistant SKOV3 and HeyA8, and the efficiency of circ_0061140 knockdown was presented in Fig. 2A. si-circ_0061140#2 was chosen for subsequent study based on its more high efficiency. Subsequently, it was found that circ_0061140 knockdown reduced the IC_50_ of PTX in PTX-resistant SKOV3 and HeyA8 cells (Fig. 2B and C), which suggested that circ_0061140 knockdown could improve PTX sensitivity in PTX-resistant SKOV3 and HeyA8 cells. Also, circ_0061140 silencing repressed cell proliferation and cell colony-forming ability in PTX-resistant SKOV3 and HeyA8 cells (Fig. 2D-F). Additionally, flow cytometry assay displayed that cell apoptosis was promoted after circ_0061140 knockdown in PTX-resistant SKOV3 and HeyA8 cells (Fig. 2G). Transwell assay showed that the migration and invasion of SKOV3/PTX and HeyA8/PTX cells were inhibited after circ_0061140 silencing (Fig. 2H and I). These findings demonstrated that circ_0061140 knockdown could repress tumorigenesis and improve PTX sensitivity in ovarian cancer in vitro.

### Circ_0061140 was associated with miR-136

**Fig. 3:**
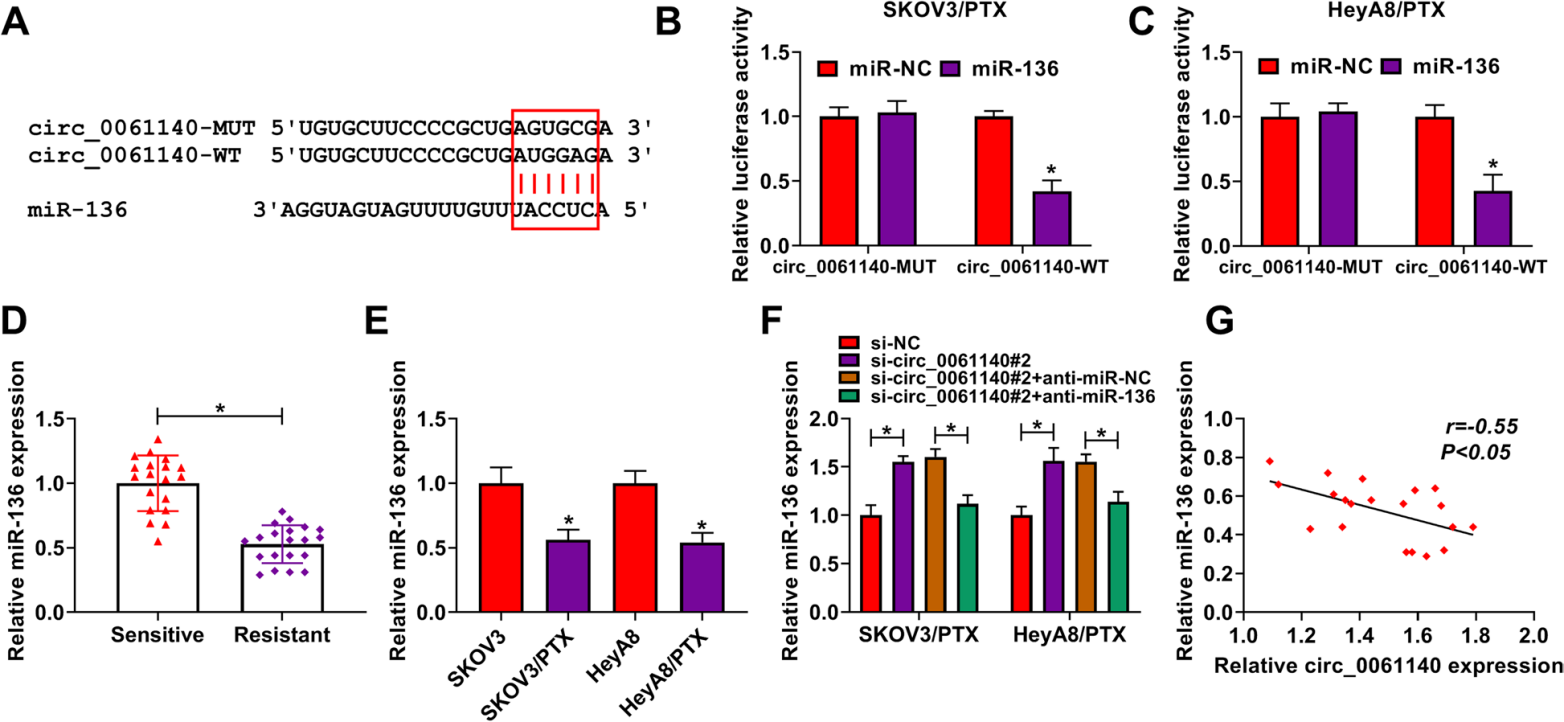
Circ_0061140 was a sponge of miR-136. (**A**) The binding sites between circ_0061140 and miR-136 were predicted by interactome online database. (**B** and **C**) Dual-luciferase reporter assay was performed to detect luciferase activity in PTX-resistant SKOV3 and HeyA8 cells. (**D** and **E**) MiR-136 expression was detected by qRT-PCR in PTX-resistant ovarian cancer tissues (*N* = 20), PTX-sensitive ovarian cancer tissues (*N* = 19), SKOV3 and HeyA8 cells as well as PTX-resistant SKOV3 and HeyA8 cells. (**F**) The impacts between circ_0061140 knockdown and miR-136 repression on miR-136 expression were revealed by qRT-PCR in PTX-resistant SKOV3 and HeyA8 cells. (**G**) Spearman correlation analysis was conducted to assess the linear relationship between circ_0061140 and miR-136 expressions. *P < 0.05

The underlying mechanisms by which circ_0061140 regulated the PTX sensitivity and cell malignancy of ovarian cancer were further explored. As shown in Fig. 3A, circ_0061140 potentially bound to miR-136. To determine the binding relationship between circ_0061140 and miR-136, we detected the efficiency of miR-136 overexpression (Figure S1A). Dual-luciferase reporter assay showed that the luciferase activity of circ_0061140-WT and miR-136 mimic group was dramatically repressed; however, there was no apparent change in circ_0061140-MUT and miR-136 mimic group in PTX-resistant SKOV3 and HeyA8 cells (Fig. 3B and C). Subsequently, the expression level of miR-136 was detected in PTX-resistant ovarian cancer tissues and cells, and results displayed that miR-136 expression was notably downregulated in PTX-resistant ovarian cancer tissues as well as SKOV3/PTX and HeyA8/PTX cells relative to control groups (Fig. 3D and E). Besides, qRT-PCR analysis determined the high efficiency of miR-136 RNAi in reducing miR-136 expression (Figure S1A). QRT-PCR results also showed that circ_0061140 knockdown strikingly increased miR-136 expression, whereas this effect was attenuated by miR-136 RNAi (Fig. 3F). Furthermore, miR-136 was negatively related to circ_0061140 expression (Fig. 3G). These data demonstrated that circ_0061140 was a sponge of miR-136 and might regulate the progression and PTX sensitivity of ovarian cancer by binding to miR-136.

### Circ_0061140 knockdown repressed the progression and PTX resistance of ovarian cancer by sponging miR-136

**Fig. 4:**
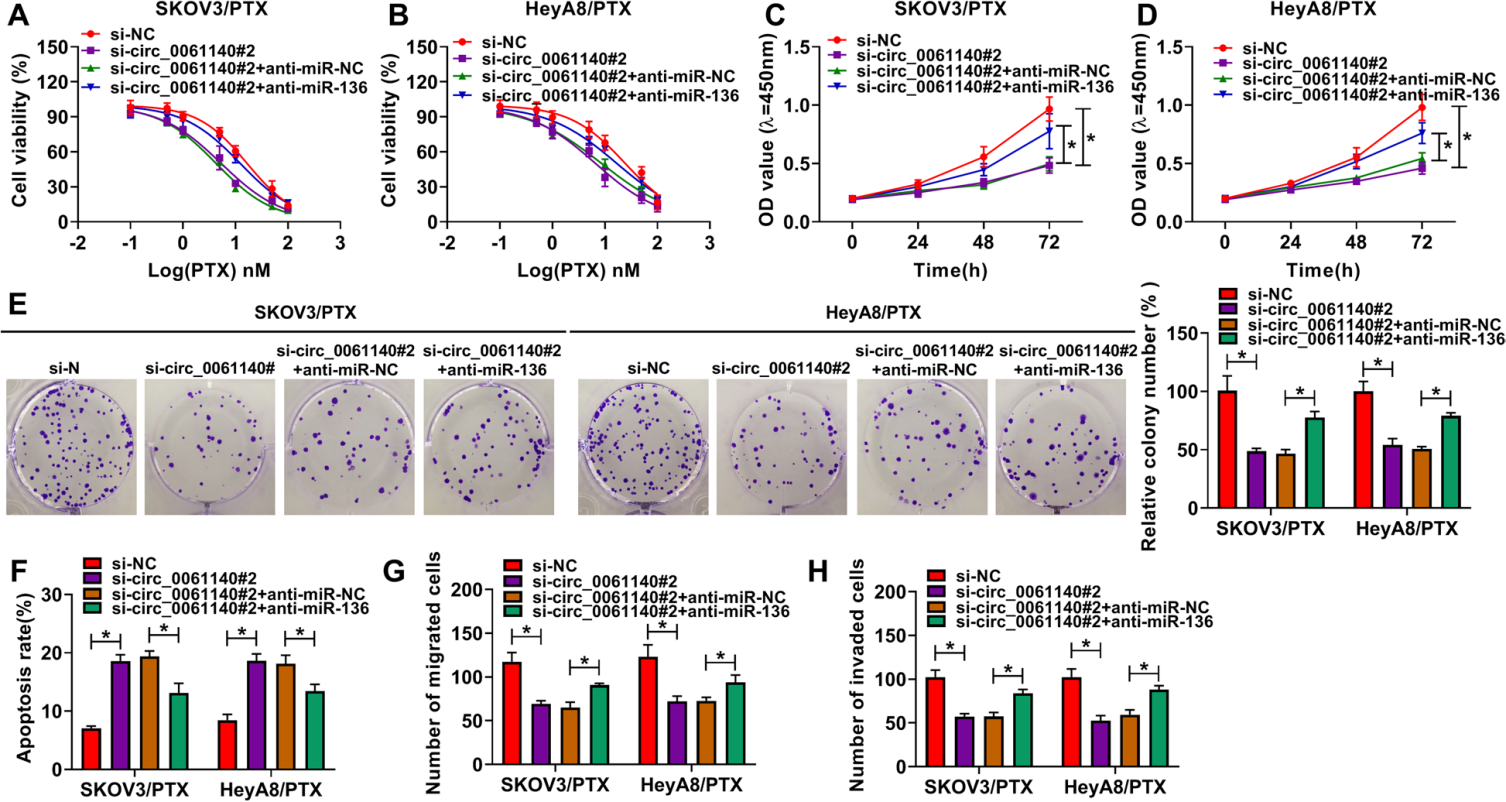
Circ_0061140 regulated the progression and PTX sensitivity of ovarian cancer by sponging miR-136. (**A** and **B**) The impacts between circ_0061140 knockdown and miR-136 depletion on cell viability after PTX treatment were revealed by MTT assay in PTX-resistant SKOV3 and HeyA8 cells. (**C**-**E**) The influences between circ_0061140 knockdown and miR-136 silencing on the proliferation of PTX-resistant SKOV3 and HeyA8 cells were explained by CCK-8 and colony formation assays. (**F**) Flow cytometry analysis was carried out to investigate the effects between circ_0061140 knockdown and miR-136 downregulation on the apoptosis of PTX-resistant SKOV3 and HeyA8 cells. (**G** and **H**) Transwell assay was performed to investigate the influences between circ_0061140 silencing and miR-136 RNAi on cell migration and invasion in PTX-resistant SKOV3 and HeyA8 cells. *P < 0.05

Whether circ_0061140 regulated PTX sensitivity and progression of ovarian cancer via miR-136 was revealed in this part. MTT assay showed that miR-136 RNAi abolished the inhibitory effect of circ_0061140 silencing on the IC_50_ value of PTX in PTX-resistant SKOV3 and HeyA8 cells (Fig. 4A and B). MiR-136 RNAi also hindered the inhibitory impacts of circ_0061140 knockdown on cell proliferation and colony-forming ability in PTX-resistant SKOV3 and HeyA8 cells (Fig. 4C-E). In addition, flow cytometry analysis showed that circ_0061140 silencing induced cell apoptosis, whereas miR-136 RNAi restored this effect (Fig. 4F). Furthermore, the migration and invasion of PTX-resistant SKOV3 and HeyA8 cells were suppressed by circ_0061140 silencing, but miR-136 RNAi impaired these influences (Fig. 4G and H). Thus, these results demonstrated that circ_0061140 modulated the progression and PTX sensitivity of ovarian cancer by associating with miR-136.

### MiR-136 was associated with CBX2 in PTX-resistant SKOV3 and HeyA8 cells

**Fig. 5:**
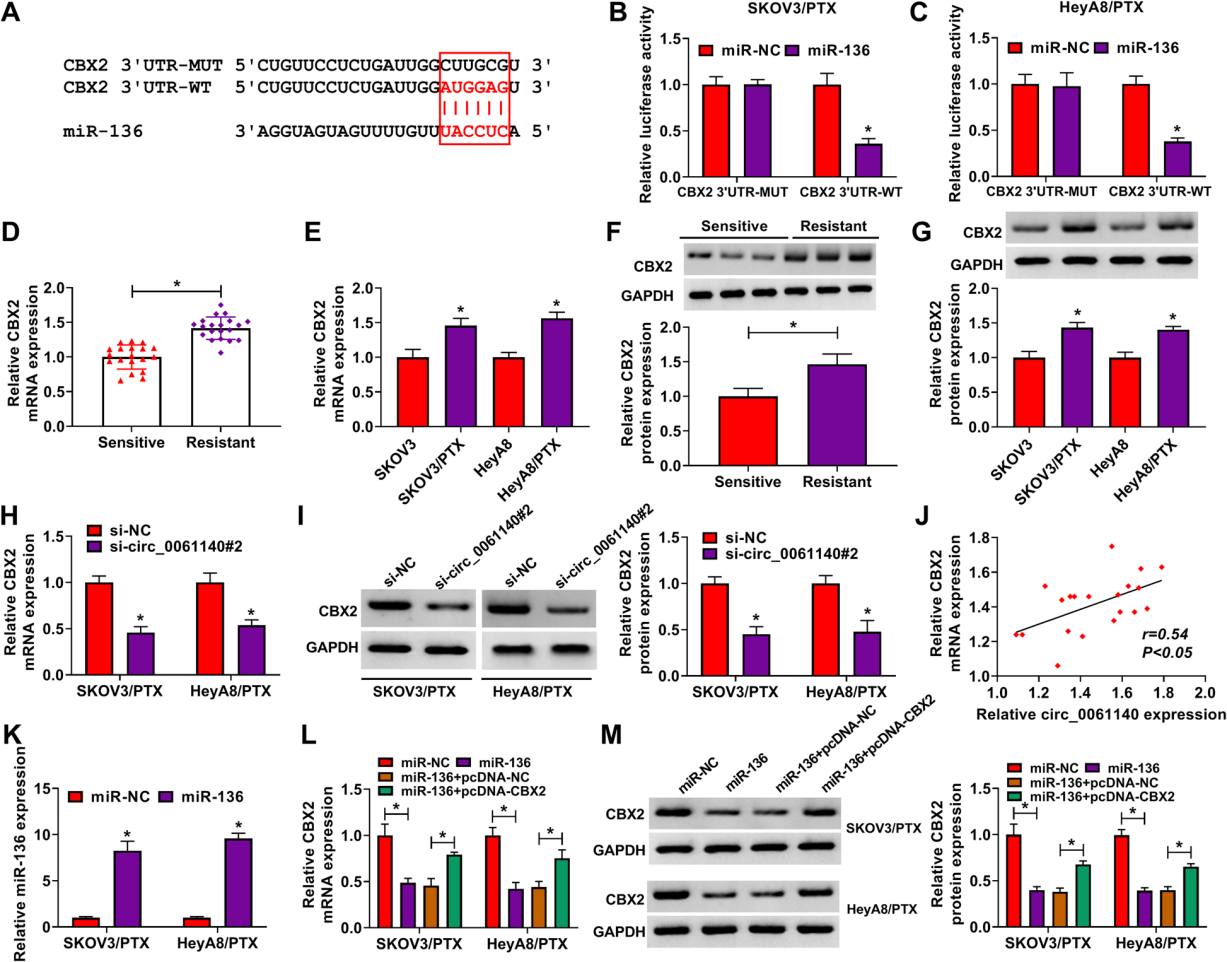
MiR-136 bound to CBX2 in PTX-resistant SKOV3 and HeyA8 cells. (**A**) Starbase online database was employed to predict the binding sites between miR-136 and CBX2. (**B** and **C**) Luciferase activity was detected by dual-luciferase reporter assay in PTX-resistant SKOV3 and HeyA8 cells. (**D**-**G**) The mRNA and protein expressions of CBX2 were detected by qRT-PCR and western blot in PTX-resistant ovarian cancer tissues (N = 20), PTX-sensitive ovarian cancer tissues (N = 19), SKOV3 and HeyA8 cells as well as PTX-resistant SKOV3 and HeyA8 cells. (**H** and **I**) The effects of circ_0061140 knockdown on the mRNA and protein expressions of CBX2 were demonstrated by qRT-PCR and western blot, respectively, in PTX-resistant SKOV3 and HeyA8 cells. (**J**) Spearman correlation analysis was conducted to elucidate the linear relationship between CBX2 and circ_0061140 expressions. (**K**-**M**) The influences between miR-136 mimic and CBX2 on the mRNA and protein expressions of CBX2 were demonstrated by qRT-PCR and western blot, respectively, in PTX-resistant SKOV3 and HeyA8 cells. *P < 0.05

The target gene of miR-136 was further sought. As predicted by starbase online database, CBX2 3’UTR contained the binding sites of miR-136 (Fig. 5A). Dual-luciferase reporter assay showed that the luciferase activity of CBX2 3’UTR-WT and miR-136 group was dramatically inhibited in PTX-resistant SKOV3 and HeyA8 cells, whereas there was no prominent change in CBX2 3’UTR-MUT and miR-136 group (Fig. 5B and C). Subsequently, qRT-PCR results showed that CBX2 mRNA expression was dramatically upregulated in PTX-resistant ovarian cancer tissues and PTX-resistant SKOV3 and HeyA8 cells as compared to control groups (Fig. 5D and E). Western blot data also displayed the protein expression of CBX2 was obviously increased in PTX-resistant ovarian cancer tissues and SKOV3/PTX and HeyA8/PTX cells as compared to control groups (Fig. 5F and G). In addition, circ_0061140 knockdown repressed the mRNA and protein expressions of CBX2 in PTX-resistant SKOV3 and HeyA8 cells (Fig. 5H and I). CBX2 expression was positively related to circ_0061140 expression (Fig. 5J). Furthermore, our data confirmed that CBX2 mRNA and protein expressions were notably downregulated after miR-136 transfection, whereas these effects were attenuated by ectopic CBX2 expression (Fig. 5K-M). These data suggested that miR-136 was associated with CBX2 in ovarian cancer cells.

### MiR-136 repressed the progression and PTX resistance of ovarian cancer by binding to CBX2

**Fig. 6:**
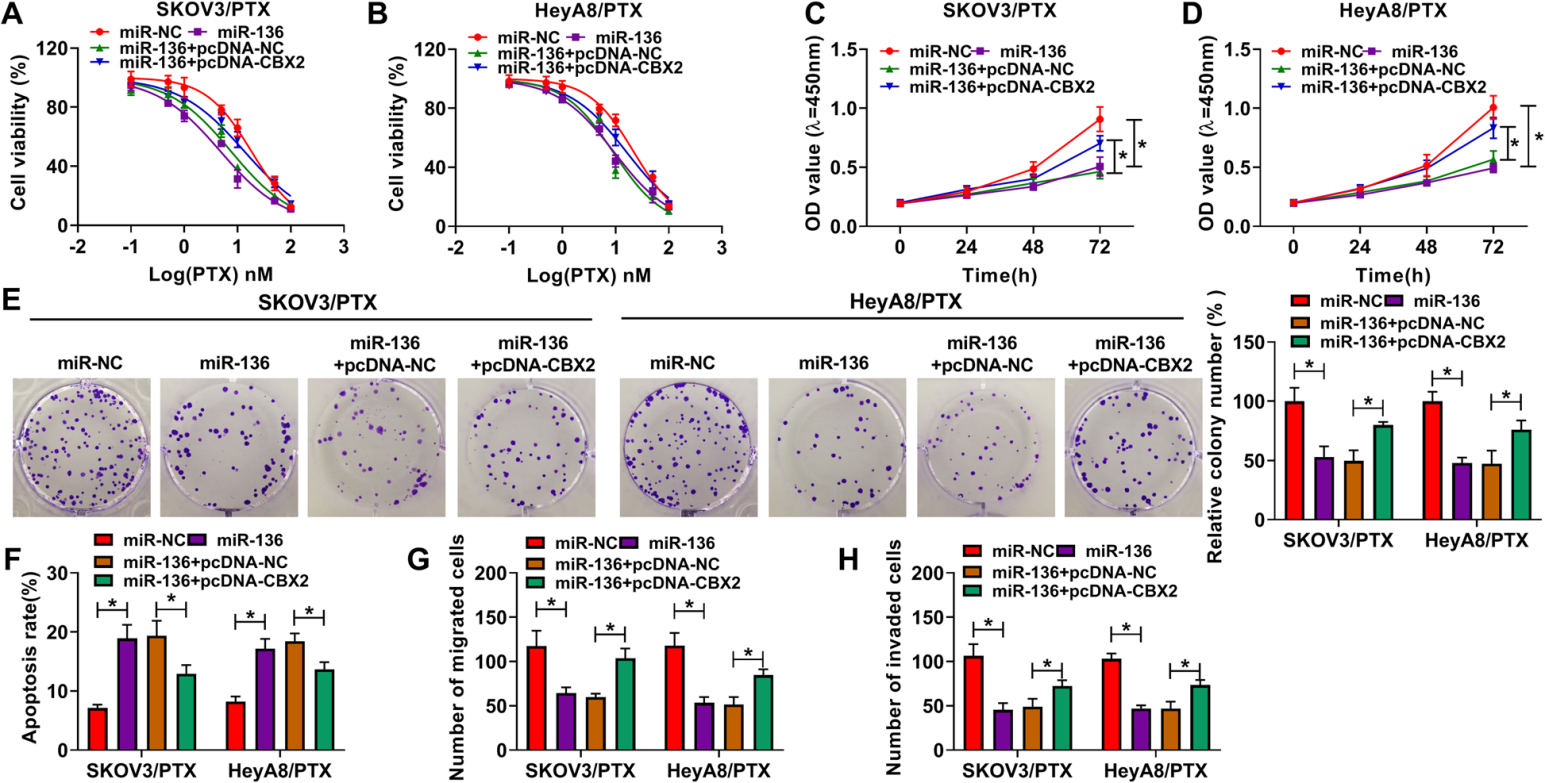
MiR-136 suppressed cell proliferation, migration and invasion, and improved cell sensitivity to PTX and induced cell apoptosis by binding to CBX2 in ovarian cancer. (**A** and **B**) The impacts between miR-136 and CBX2 on the IC_50_ value of PTX were determined by MTT assay in PTX-resistant SKOV3 and HeyA8 cells. (**C**-**E**) The effects between miR-136 and CBX2 overexpression on the proliferation of PTX-resistant SKOV3 and HeyA8 cells were determined by CCK-8 and colony formation assays. (**F**) Flow cytometry analysis was performed to investigate the influences between miR-136 and CBX2 on the apoptosis of PTX-resistant SKOV3 and HeyA8 cells. (**G** and **H**) Transwell assay was carried out to investigate the effects between miR-136 and CBX2 on the migration and invasion of PTX-resistant SKOV3 and HeyA8 cells. *P < 0.05

Further, the regulatory relationship of miR-136 and CBX2 in the progression and PTX sensitivity of ovarian cancer was explored. The overexpression efficiency of CBX2 was firstly detected by western blot and result was shown in Figure S1B. MTT assay showed that MiR-136 mimic decreased the IC_50_ value of PTX, whereas this effect was restored by CBX2 overexpression (Fig. 6A and B). CCK-8 and colony formation assays also demonstrated that the proliferative and colony-forming abilities of PTX-resistant SKOV3 and HeyA8 cells were repressed by miR-136 mimic; however, these impacts were attenuated after CBX2 overexpression (Fig. 6C-E). In addition, results showed that miR-136 induced cell apoptosis, but ectopic CBX2 expression relieved this effect (Fig. 6F). MiR-136 overexpression suppressed cell migration and invasion in PTX-resistant SKOV3 and HeyA8 cells, but these impacts were attenuated by CBX2 (Fig. 6G and H). These findings manifested that miR-136 could regulate ovarian cancer sensitivity to PTX and progression by binding to CBX2.

### Circ_0061140 knockdown inhibited tumor growth and PTX resistance in ovarian cancer in vivo

**Fig. 7:**
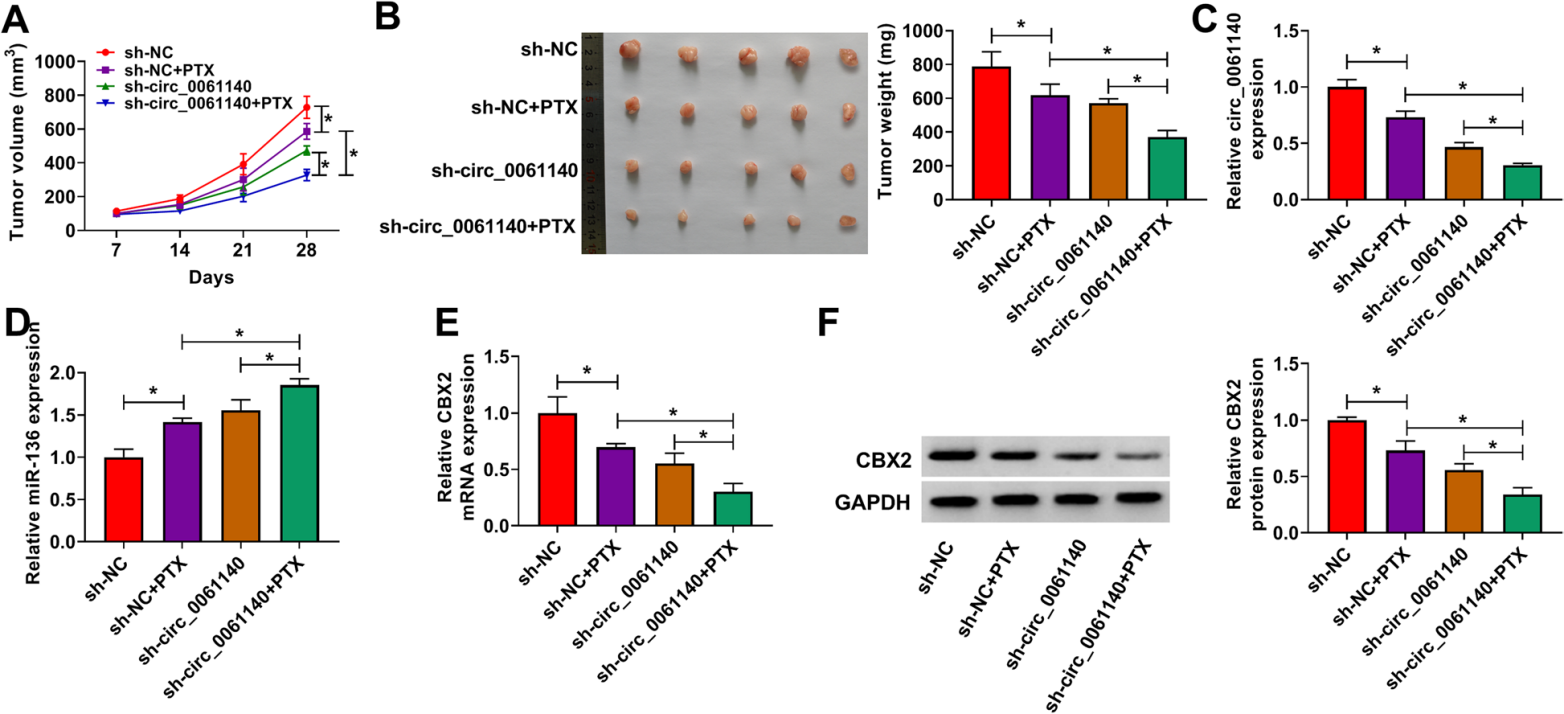
Circ_0061140 silencing enhanced the inhibitory effects of PTX on tumor growth in vivo. (**A** and **B**) The effects of circ_0061140 absence on the volume and weight of tumors after PTX treatment were revealed. (**C** and **D**) QRT-PCR was employed to determine the impacts of circ_0061140 silencing on the expressions of circ_0061140 and miR-136 under PTX exposure in vivo. (**E** and **F**) The mRNA and protein expressions of CBX2 were uncovered by qRT-PCR and western blot, respectively, in forming tumors from sh-NC, sh-NC + PTX, sh-circ_0061140 or sh-circ_0061140 + PTX group. *P < 0.05

The effects of circ_0061140 silencing on the PTX sensitivity and progression of ovarian cancer in vivo were further analyzed. Results showed that PTX treatment dramatically repressed the volume and weight of tumors, whereas these effects were promoted by circ_0061140 knockdown (Fig. 7A and B). In addition, PTX treatment significantly downregulated circ_0061140 expression, and circ_0061140 knockdown enhanced this effect (Fig. 7C). Then, results testified that miR-136 expression was obviously upregulated after PTX treatment, and circ_0061140 absence facilitated this impact (Fig. 7D). The mRNA and protein expressions of CBX2 were apparently decreased by PTX, whereas these influences were promoted by circ_0061140 silencing (Fig. 7E and F). These results demonstrated that circ_0061140 could mediated PTX sensitivity and tumor formation by modulating miR-136 and CBX2 expression in vivo.

## Discussion

Numerous studies have showed that circRNA is involved in the progression of cancers, including ovarian cancer [11, 24]. PTX-based chemotherapy is the first-line therapy for ovarian cancer; however, drug resistance brings a heavy burden to the chemotherapy. CircRNA has been reported to regulate drug resistance in lots of cancers [25]. Nevertheless, the effect of circ_0061140 on the sensitivity of ovarian cancer to PTX is still indistinct. In this study, we explored circ_0061140 role in PTX sensitivity of ovarian cancer, and our data showed that circ_0061140 knockdown repressed PTX resistance.

CircRNAs can regulate PTX sensitivity in many cancers. As reported, circ-ring finger protein 111 (circ-RNF111) and circ-ABCB10 enhanced PTX resistance in breast cancer [26, 27]. Circ_PVT1 repressed gastric cancer resistance to PTX via binding to miR-124-3p [28]. Circ_BIRC6 overexpression relieved PTX-induced repression of hepatocellular carcinoma progression through trapping miR-877-5p [29]. Additionally, circ_ZFR promoted PTX resistance in lung cancer in a miR-195-5p-dependent manner [30]. Circ_0061140 has been identified as a tumor promoter in bladder cancer [31], endometrial carcinoma [32] and prostate cancer [33]. In this paper, results showed that circ_0061140 silencing decreased the IC_50_ value of PTX. Additionally, circ_0061140 was dramatically increased in PTX-resistant ovarian cancer tissues and cells. Circ_0061140 knockdown impaired cell proliferation, migration and invasion, whereas accelerated cell apoptosis. Furthermore, circ_0061140 absence repressed tumor formation and promote PTX sensitivity in ovarian cancer in vivo. The above data demonstrated that circ_0061140 acted as a tumor promoter and suppressed PTX sensitivity in PTX-resistant ovarian cancer.

Based on that circRNA regulated gene expression via absorbing miRNA, the miRNAs able to combine with circ_0061140 were predicted and identified. Results showed that circ_0061140 acted as a sponge of miR-136. Previous study showed that circ_0023404 facilitated tumor development by sequestering miR-136 in cervical cancer [34]. MiR-136 inhibited cell viability and proliferative ability in ovarian cancer [35]. In addition, investigators testified that miR-136 introduction contributed to the sensitivity of ovarian cancer to PTX by binding to notch receptor 3 (NOTCH3) [35]. In this study, we found miR-136 was lowly expressed in PTX-resistant ovarian cancer tissues and cells, and rescue experiments showed that miR-136 RNAi attenuated the effects of circ_0061140 knockdown on tumor progression and PTX sensitivity. These evidences indicated that miR-136 improved PTX sensitivity and hindered tumor process in PTX-resistant ovarian cancer. Meanwhile, these findings also demonstrated that circ_0061140 regulated the PTX sensitivity and progression of ovarian cancer cells via absorbing miR-136.

Accumulating studies have suggested the importance of CBX members in cancer development [36]. As a component of polycomb repressive complex 1 (PRC1), CBX family includes CBX1-CBX8 [21]. Based the prediction of bioinformatics tools, we found that miR-136 contained the binding sites of CBX2, CBX3 and CBX4. Subsequent analysis revealed that miR-136 negatively regulated CBX2 and CBX4 expressions (data not shown). CBX2 plays a vital role in transcriptional regulatory process and its expression level is high in normal ovarian tissues [37, 38]. Also, Dou et al. also explained that CBX2 contributed to cell proliferation and metastasis in ovarian cancer [38]. Compared with the limited data on the role of CBX4 in ovarian cancer, CBX2 was employed as the potential target gene of miR-136. In this paper, dual-luciferase reporter assay proved that CBX2 was associated with miR-136. Additionally, qRT-PCR and western blot analysis showed that CBX2 was overexpressed in PTX-resistant tissues and cells. Functional experiments also displayed that CBX2 overexpression restored the effects of miR-136 mimic on tumor progression and PTX sensitivity, which demonstrated that CBX2 acted as an oncogene and could inhibit PTX sensitivity in PTX-resistant ovarian cancer. Meanwhile, these data also showed that miR-136 regulated the sensitivity of ovarian cancer to PTX and tumor progression by targeting CBX2.

There were some shortcomings that needed to be considered when evaluating our findings. Published data reported that miR-136 inhibited CBX4 expression in renal cell carcinoma tissues [39] and cervical cancer cells [40], suggesting that miR-136 might regulate ovarian cancer cell malignancy by binding to CBX4. The hypothesis would be explained in future. Besides, although tumor growth and response to treatment could be easily observed when studying cancer cell malignancy, the in vivo animal model could not mimic the environment for cancer cells to grow and migrate.

## Conclusions

All in all, circ_0061140 expression was dramatically increased in PTX-resistant ovarian cancer tissues and cells. Circ_0061140 absence hindered cell proliferation, migration and invasion, and improved PTX sensitivity and accelerated cell apoptosis via downregulating CBX2 expression through binding to miR-136 in PTX-resistant ovarian cancer in vitro. Furthermore, circ_0061140 silencing improved PTX sensitivity in ovarian cancer in vivo. Our findings suggested that circ_0061140 might act as a prospective target for further studying ovarian cancer treatment with PTX.

## Supplementary Information

**Additional file 1: Table S1.** Primer sequences used in qRT-PCR.

**Additional file 2: Table S2** The clinicopathologic features of ovarian cancer patients in this study.

**Additional file 3: Figure S1** The efficiency of miR-136 overexpression, miR-136 knockdown and CBX2 overexpression was detected by qRT-PCR or western blot in PTX-resistant SKOV3 and HeyA8 cells (A and B). *P < 0.05.

## Data Availability

The analyzed data sets generated during the study are available from the corresponding author on reasonable request.
