# Supplementary material for: Circ_0061140 knockdown inhibits tumorigenesis and improves PTX sensitivity by regulating miR-136/CBX2 axis in ovarian cancer
Source: J Ovarian Res. 2021 Oct 14;14:136. doi: 10.1186/s13048-021-00888-9 (PMC8518226; doi:10.1186/s13048-021-00888-9)
Supplement: Supplementary file 1 — Additional file 1: Table S1. Primer sequences used in qRT-PCR. [file 13048_2021_888_MOESM1_ESM.docx]

| Gene | Sequences of primers (From 5’ to 3’) |
| --- | --- |
| circ_0061140 Forward | AGAAGTGCGGAATAGGTT |
| circ_0061140 Reverse | CCAGTTTGGGTGTTGACT |
| miR-136 Forward | ACTCCATTTGTTTTGATGATGGA |
| CBX2 Forward | GGAACTGCACCAGATGCTCA |
| CBX2 Reverse | CAATGCATCGTCAGAAGTGCC |
| GAPDH Forward | GGAGCGAGATCCCTCCAAAAT |
| GAPDH Reverse | GGCTGTTGTCATACTTCTCATGG |
| U6 Forward | CTCGCTTCGGCAGCACATA |
| U6 Reverse | CGAATTTGCGTGTCATCCT |

**Supplementary Table 1. Primer sequences used in qRT-PCR**
