# Supplementary material for: Circ_0061140 knockdown inhibits tumorigenesis and improves PTX sensitivity by regulating miR-136/CBX2 axis in ovarian cancer
Source: J Ovarian Res. 2021 Oct 14;14:136. doi: 10.1186/s13048-021-00888-9 (PMC8518226; doi:10.1186/s13048-021-00888-9)
Supplement: Supplementary file 2 — Additional file 2: Table S2 The clinicopathologic features of ovarian cancer patients in this study. [file 13048_2021_888_MOESM2_ESM.docx]

**Table 1.** **The clinicopathologic features in patients with** **ovarian cancer in this study**

| Parameters | N=39 |
| --- | --- |
|  |  |
| Age,years |  |
| <60 | 16 |
| ≥60 | 23 |
| Tumor size |  |
| <4 cm | 22 |
| ≥4 cm | 17 |
| Histological classification |  |
| Serous | 26 |
| Mucinous | 13 |
| FIGO stage |  |
| < III | 24 |
| ≥III | 15 |
| Lymph node metastasis |  |
| Yes | 14 |
| No | 25 |
| Chemosensitivity |  |
| PTX-sensitive | 19 |
| PTX-resistant | 20 |
