# Supplementary figures and images for: Circ_0061140 knockdown inhibits tumorigenesis and improves PTX sensitivity by regulating miR-136/CBX2 axis in ovarian cancer
Source: J Ovarian Res. 2021 Oct 14;14:136. doi: 10.1186/s13048-021-00888-9 (PMC8518226; doi:10.1186/s13048-021-00888-9)

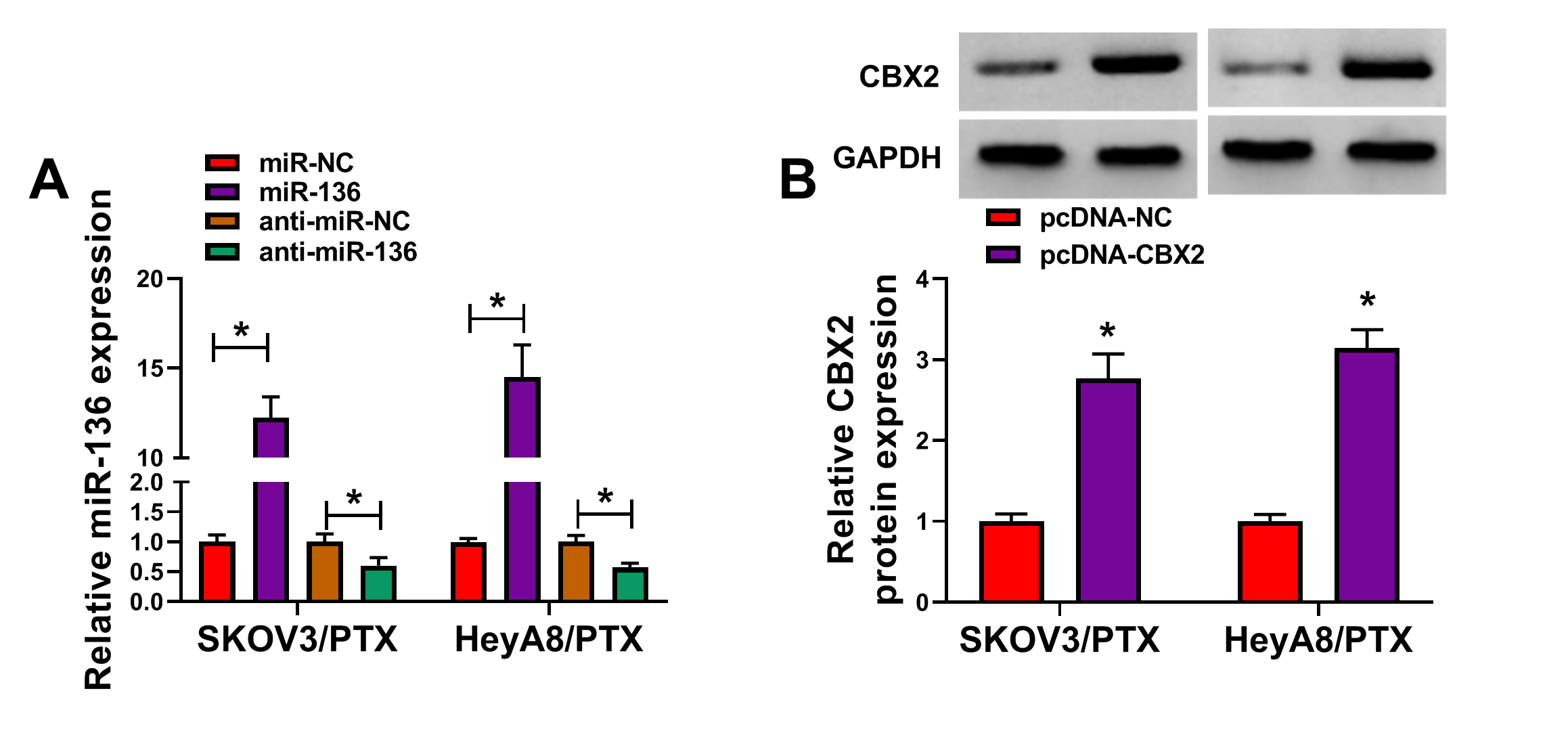

Supplement: Supplementary file 3 — Additional file 3: Figure S1 The efficiency of miR-136 overexpression, miR-136 knockdown and CBX2 overexpression was detected by qRT-PCR or western blot in PTX-resistant SKOV3 and HeyA8 cells (A and B). *P < 0.05. [file 13048_2021_888_MOESM3_ESM.tif]
